# Strangulated Hernia

**Published:** 1861-10

**Authors:** W. A. Gordon

**Affiliations:** Wausau, Wis.


ARTICLE III.

### STRANGULATED HERNIA.

By W. A. GORDON, M. D., Wausau, Wis.

In the month of September, A. D. 1858, I was called eight miles distant, to see Mr. McC., said to have been attacked with bilious fever. Upon my arrival, I found a young man (farmer,) aged 24 years, of sanguine temperament, and a vigorous constitution. He had been taken suddenly ill three days previous to my visit, with very severe pains in his bowels; slight nausea and vomiting, with a considerable degree of fever. Symptoms now present were, quick, full pulse, 124 per minute; epigastric region and abdomen somewhat tender; frequent vomiting; great thirst and general lassitude. I ascertained that there had been no fecal evacuation for the past few days. Pills and castor oil had been given, but with no other effect than increasing the irritability of the stomach. A brief investigation convinced me that I had something more to deal with, than a common remittent fever, and the circumstances of the case led me to suspect a hernia. But upon further inquiry, I could elicit nothing from the patient to corroborate my opinion. I must confess that I was not a little embarrassed upon receiving such information, and was not satisfied to reveal my diagnosis to the anxious friends, until I was thoroughly convinced as to the true nature of the case. I proposed an examination, and accordingly passed my hand over the inguinal region and scrotum, and as I had previously anticipated, found considerable enlargement of the left groin, and corresponding testicle. I expressed some astonishment to the patient that he had remained ignorant of this swelling during his illness; but said he, "It has been there these ten years." Judging from his statement of the usual size of the tumor, it was now considerably enlarged; not sufficiently so, however, to attract his attention previous to my examination, which revealed a case of oblique inguinal hernia. From its nature and long standing, I concluded that the original hernia was protrusion of the omentum, and the cause of present difficulty was produced by a portion of intestine being thrust forward in the same channel, induced by powerful action of the abdominal muscles and diaphragm, in the act of loading hay on the day previous to his illness.

The bladder was evacuated by means of the catheter, (there having been no spontaneous flow for twelve hours,) when I proceeded in the usual manner to reduce by the taxis. After manipulating about twenty minutes, the tumor was reduced nearly one-third in size; and any further endeavors at reduction seemed unavailing. It now occurred to me that perhaps the new protrusion was reduced, as the patient expressed himself as feeling relieved, and thought that the tumor was about its usual size. I ordered the patient to roll on either side, at the same time keeping the pelvis a little elevated, hoping that the gravity of the viscera would entirely retract the intestine from the canal, providing the hernia was not already reduced.

I remained with the patient about two hours, when he seemed so comfortable, I ordered the following enema:

℞ Chlor. sodium,

Sap. cast., - - - - āā ʒj.

Aqua fervent, - - - Oj.

One half to be used at the temperature of the body, and if no evacuation in two hours, administer the remainder. Hop fomentations in the meantime were applied to the abdomen. I left my patient comparatively easy, and with sanguine hope of finding him next day in a fair way for recovery. It was twenty-four hours before I was again able to see him, when to my sad disappointment, I found every symptom aggravated.

He had clandestinely been walking about his room, and re-adjusted the compress and bandage to suit his own liking. I now requested counsel, and forthwith dispatched a messenger for Dr. S. Marks, of Steven's Point, a distance of fifty miles, at that time the nearest reliable counsel. In the meantime, large doses of opium were given with the view of obtaining its specific effects, hoping we might yet succeed without an operation. It was full eighteen hours before the doctor arrived; and when we repaired to the residence of the patient, it was only to find him in a still more unfavorable condition. But little of the opium had been retained, and there was now more or less vomiting of bile, and stercoraceous matter; scanty urine; abdomen somewhat tense and painful; quick, wiry pulse, and a great restlessness and prostration. Attempts were again made to reduce by the taxis, as the tumor was much larger than when I last saw the patient. Our efforts seemed quite effectual in reducing its size; and from the relief which it afforded, we arrived at the conclusion that the hernia was again reduced, and applied dressings accordingly. Ordered hop fomentations to the bowels and tumor, and after six hours, if the patient was comfortable, then clysters as I had previously ordered.

We remained with the patient during the night, and at five o'clock A. M., a copious evacuation took place, containing quite a quantity of fecal matter. Vomiting had very nearly abated during the night, and as the patient expressed himself in the morning, "he felt better all over." His pulse was now 100 per minute; quite soft and comfortable; less tenderness of the abdomen, and no vomiting for four hours previous to the discharge. We examined the tumor before leaving; and anticipating no further trouble on the part of our patient, left him the following:

℞ Nit. potassa.
Pulv. doveri, - - - āā ʒj.
Hyd. submur. - - - grs. x.
M. div. in pulv. no. vj.

One to be given every four hours. This prescription was to be followed by a mild dose of castor oil.

Upon my arrival home, I was called in an opposite direction, and was unexpectedly detained, so that it was quite impossible for me to again visit the patient for thirty-six hours. I had received two dispatches from there during this time, and upon my arrival, I was not surprised that the friends had been alarmed. The castor oil had been rejected, and from that time he had been vomiting, which was now superceded by hiccough; quick feeble pulse; cold sweat, and a decided hippocratic face.

To again send for counsel, which would consume, at least, ten hours, rendered the chances of the patient, (if he had any,) entirely hopeless; and on the other hand, to operate alone, and have the patient die under the operation, was indeed anything but a pleasant thought. It was apparent that he could live but a few hours at most, and the chances for him to survive the operation were extremely doubtful. After a few moments' consultation with the friends, it was decided that the operation should be immediately performed. With no other assistant than a neighboring farmer, the patient was anæsthetized and the operation commenced, by making an incision along the axis of the tumor about four inches in length, commencing mid-way between the internal and external rings, and extending to the middle of the scrotum. The coverings were successively raised with the forceps, and the apex of the elevated portion, slightly nicked, to facilitate the introduction of the director, which guided the bistoury in cutting through the different layers to the extent of about two inches, when arriving at the sac, it was found firmly attached by means of old adhesions on all sides.

It was evident that more or less dissection was necessary, and the coverings were now cut through to the full extent of the original incision. About three ounces of serous fluid had gravitated to the lower extremity of the tumor, affording a good point for opening the sac. The omentum was protruded about three inches through the external ring, indurated and surrounding the spermatic cord about two inches. It was very much distended with gas, and presented a dark purple color. Fortunately we succeeded in nicely separating the gut from the sac, but in dissecting up the diseased omentum and detaching it from the spermatic vessels, it was unavoidably mutilated, so that it became necessary to cut away about two and a half inches. The dissection involved three small arteries, (branches of the superior mesenteric,) which required the ligature. The stricture was now divided, the intestine returned; and the portion of sound omentum to which were attached the ligatures, left in the external ring, thereby affording free exit for the discharge through the external wound, which was dressed with interrupted suture and adhesive straps. Morphine was given to allay pain, and milk-punch as a stimulating and nutritious diet. This treatment was continued twenty-four hours, when the patient seemed to have rallied sufficiently to admit of the administration of a cathartic. We accordingly prescribed ol. ricini, ℥j.; ol. limon. gtts. x, which operated very kindly in about four hours.

There remained some tympanitis and tenderness of the abdomen, accompanied with great debility, for which the following was prescribed:

℞ Hyd. submur., - - - grs. xij.
Pulv. opii., - - - - grs. vj.
Quinine sulph., - - - grs. x.
M. div. in pulv. vj.

One of these powders to be given every four hours, and beef-tea for nourishment. This treatment was continued for three days, when the specific effect of the alterative manifested itself, and simultaneously the disappearance of the tenderness of the abdomen.

On the fifth day, subsequent to the operation, the wound was secreting laudable pus, and the tenth day, the ligatures were removed. The patient now improved rapidly, and six week from the time of the operation, he called at my office for the proper adjustment of his truss, which (I will here remark,) he has made no use of for the past twelve months,—feeling that he is permanently cured.

The remarkable features in connection with this case are, 1st, the recovery of the patient; and 2d, the radical cure of the hernia.

To my own mind, it was one well calculated to deceive even the more experienced surgeon; and the combined circumstances which deferred the operation until little or no hope remained, rendered the case one of more than ordinary interest.

